# Unpredictability is a childhood adversity that contributes to mental health problems

**DOI:** 10.1038/s44220-026-00681-x

**Published:** 2026-07-21

**Authors:** Laura M. Glynn, Sabrina R. Liu, Charles Golden, Michael Weiss, Candice Taylor Lucas, Dan M. Cooper, Louis Ehwerhemuepha, Hal S. Stern, Tallie Z. Baram

**Affiliations:** 1 Department of Psychology, Chapman University, Orange, CA USA; 2 Department of Psychology, California State University, San Marcos, San Marcos, CA USA; 3 Children’s Hospital of Orange County, Orange, CA USA; 4 Department of Pediatrics, University of California, Irvine, Irvine, CA USA; 5 Department of Statistics, University of California, Irvine, Irvine, CA USA; 6 Department of Anatomy and Neurobiology, University of California, Irvine, Irvine, CA USA; 7 Department of Neurology, University of California, Irvine, Irvine, CA USA

**Keywords:** Risk factors, Psychology, Diseases

## Abstract

Although adverse childhood experiences (ACEs) increase risk for mental illness at the population level, existing ACEs screens are less helpful in forecasting individual outcomes, suggesting they may not capture significant elements of childhood adversity. We have previously identified unpredictable parental and household experiences as an ACE that portends poorer cognitive and mental health. However, the contribution of unpredictability to established ACEs in real-world settings is unknown. Here, leveraging existing ACEs screening in California, we added the five-item Questionnaire on Unpredictability in Childhood (QUIC-5) in 19 pediatric clinics spanning broad sociodemographic constituencies and compared in ~30,000 children the link of each screen with mental health diagnoses. Scores on either the ACEs or QUIC-5 associated with probabilities of depression, externalizing symptoms, sleep disorders, anxiety and somatic symptoms. Each screen provided unique contributions and combining them often doubled the strength of associations. For depression and sleep disorders, the QUIC-5 identified vulnerable individuals missed by ACEs screen, improving risk detection and facilitating future interventions.

## Abstract (web summary)

In this population-based cohort study, Glynn et al. tested the contribution of childhood unpredictability to mental health outcomes in ~30,000 participants, examining the relative and additive roles of adverse childhood experiences and unpredictability as risk factors for mental health problems.

## Main

Pivotal studies, including the Centers for Disease Control and Prevention (CDC)–Kaiser Permanente study^1^ focusing on adverse childhood experiences (ACEs), documented cumulative effects of exposure to potentially traumatic experiences (for example, abuse, neglect or violence witnessing) on a wide range of mental and physical health conditions. These discoveries prompted examination of the enduring role of ACEs in health and disease^2–7^ and it is now estimated that the economic burden of exposure to ACEs in the US adult population is US$14.1 trillion annually^3^. Given the accumulating evidence regarding the human and fiscal toll of ACEs, calls to address the prevalence and consequences of ACEs are rapidly increasing^5,8–16^. At the forefront of these efforts, California became the first state (in 2020) to implement a publicly supported screening program. Although the goal of the program is to dramatically reduce the burden of ACEs on the population^8,16^, the value of this public health initiative has been questioned on several grounds, the primary being that ACEs screens identify risk at the population level, yet perform little better than chance at the level of the individual, limiting their ability to direct prevention or intervention resources to the most vulnerable children^17–21^.

There are several potential explanations for why ACEs scores are limited in their ability to better detect health risks. One possibility is that substantial sources of stress and trauma occurring in childhood are missed with existing instruments. Indeed, one early-life exposure that is not currently included in standard screening instruments is unpredictability of parental and environmental signals received by the child, which activates the brain’s stress responses^21–25^. The concept that unpredictable signals to the developing brain are stressful and disrupt brain maturation arose initially in experimental animal studies^21–25^. Since then, unpredictable parental care and lack of structure in the family and home environment have been shown to strongly predict poorer cognitive and emotional development across a broad range of cultures and sociodemographic groups^26–33^. Unpredictability in childhood, independent from parental support and sensitivity, has been linked by several independent groups to decreased executive control, a slower trajectory of cognitive development, poorer memory and increased risk for depression, anhedonia, anxiety and post-traumatic stress disorder later in life^26–32,34–38^. These associations persist after consideration of other well-established ACEs (for example poverty, abuse or neglect), suggesting that unpredictable experiences in themselves are a robust risk factor for poor mental health outcomes, and their absence from existing assessments of ACEs may account for some of the limitations of these assessments in predicting risk profiles.

Here, we test the contribution of unpredictability to mental health outcomes in a large, population-based cohort (~30,000 participants). We examine the relative and cumulative contributions of ACEs and unpredictability as risk factors for mental health problems. We leverage the existing ACEs screening implemented in the Children’s Hospital of Orange County (CHOC) primary care network^39^, engaging with families from a broad range of sociodemographic backgrounds. Adding our well-validated five-item measure of unpredictability^40^ we address the following questions: (1) When used in routine pediatric primary care, does ACEs screening with the Pediatrics ACEs and Related Life-events Screen (PEARLS) identify children at increased risk of mental health problems? (2) Does screening for parental and environmental unpredictability provide added value in assessing risk beyond ACEs screening?

## Results

### Exposures to ACEs and to unpredictability increase with age

**Fig. 1:**
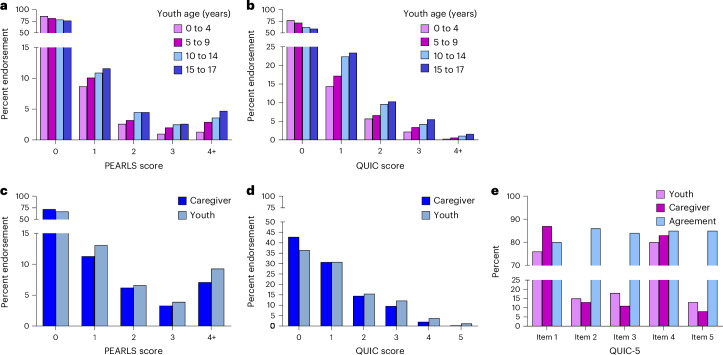
The distribution of ACEs, measured using PEARLS, and of unpredictable early-life experiences, measured using QUIC-5. **a**,**b**, The distribution of scores across child age for PEARLS (**a**) and QUIC-5 (**b**), based on the caregiver report, which is available for all ages. **c**,**d**, A comparison of the distribution of ACEs (PEARLS) (**c**) and unpredictability (QUIC-5) (**d**) for caregiver report and youth self-report, the latter for age 12 years and above. **e**, The agreement of item endorsement between youth and caregiver reports on the QUIC-5.

For all ages, 0–17 years (Table 1), older children experienced, on average, more exposure to both ACEs and unpredictability (Fig. 1a,b). Notably, exposure to at least one ACE (13%) or at least one form of unpredictability (23%) was observed even among the youngest children at ages 0–4 years. In addition, youths generally reported more exposures to both ACEs and unpredictability than did caregivers (Fig. 1c,d). The correlation between youth and caregiver reports was 0.64 for PEARLS and 0.54 for the five-item Questionnaire on Unpredictability in Childhood (QUIC-5) (*P* < 0.001 for both). Further evidence of the validity of the QUIC-5 screen is provided by the strong agreement of specific item endorsement between youth and caregiver ranging from 0.8 to 0.85. (Fig. 1e). Finally, scores on the two screens were associated: the correlation between QUIC-5 and PEARLS was 0.51 for child report and 0.43 for caregiver report (*P* < 0.001 for both).

**Table 1:** Participant characteristics

	*n*	Percent (%)
Child age (years)		
0–4	5,917	19.8
5–9	8,165	27.3
10–14	9,387	31.4
15–17	6,285	21.0
Child race		
Asian	2,941	9.8
Black	512	1.7
Native American or Alaskan	114	0.4
Native Hawaiian	23	0.1
White	7,534	25.2
More than one race or other	9,323	31.2
Unknown	9,414	31.5
Child ethnicity		
Hispanic/Latino/a	17,472	58.5
Not Hispanic/Latino/a	8,903	29.8
Unknown	3,486	11.7
Preferred language		
English	24,764	82.9
Spanish	5,043	16.9
Child gender		
Female	14,418	48.3
Male	15,443	51.7
Insurance		
Public	20,359	68.2
Private	9,340	31.3
Self or other	162	0.5

Demographic data were retrieved from electronic health records. Race, ethnicity and gender were collected from the caregiver at initial registration. Preferred language indicates the language in which the screens were administered. Insurance status was determined from the visit concurrent with screening.

### Both ACEs and unpredictability screens portend mental health and somatic symptoms

**Fig. 2:**
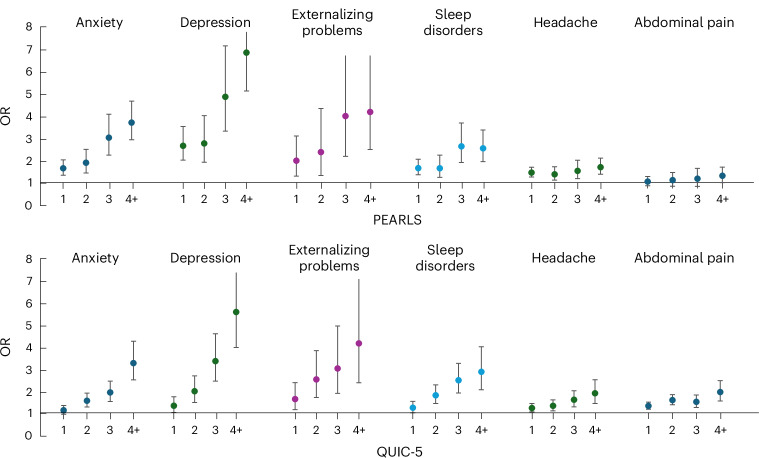
Both ACEs (PEARLS; *n* = 8,906) and unpredictability (QUIC-5; *n* = 9,870) screens associate with child mental health and somatic symptoms outcomes. The figure depicts results from the youth self-report. ORs and 95% CIs are shown. CIs that do not cross the line indicate a statistically significant increased risk (see Supplementary Tables 1 and 2 for the ORs and CIs for both youth and caregiver).

Increasing exposures to ACEs, assessed with PEARLS, and to unpredictability, measured with QUIC-5, associated with a higher probability of a mental health problem (depression, anxiety, externalizing problems and sleep disorder) and somatic symptoms (abdominal pain and headache). This was true for reports by both youth (Fig. 2) and caregivers. Across diagnoses, the associations were generally dose-dependent, with odds ratios (ORs) increasing with each additional exposure (ORs and confidence intervals (CIs) are provided for both reporters for all outcomes in Supplementary Tables 1 and 2). For youth self-report, the ORs for those with 4 or more exposures compared with those with zero ranged from 1.7 to 7.2 for PEARLS and 1.6 to 6.1 for QUIC-5. The range of ORs for caregiver reports was similar in magnitude. Running the same analyses with adjustments for private versus public insurance (a proxy for socioeconomic status) did not substantively alter these associations (Supplementary Tables 3 and 4).

### Measuring unpredictability uncovers children at risk for mental health problems that are not detected by ACEs screens

**Fig. 3:**
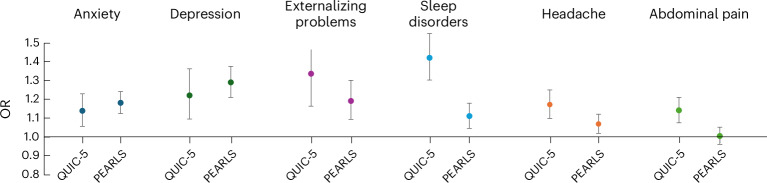
Youth self-reports on ACEs (PEARLS) and unpredictability (QUIC-5) screens independently predict child health outcomes in logistic regression model including both as predictors (*n* = 7,149). The results are similar for caregiver report. ORs and 95% CIs are shown (see Supplementary Tables 5 and 6 for numerical values). CIs that do not cross the line indicate statistically significant increased risk.

The correlation between PEARLS and QUIC-5 scores raised the question of whether including screening for unpredictability adds any value for predicting child health outcomes beyond that obtained from PEARLS alone. To address this question, we conducted two types of analyses. First, we determined the adjusted ORs for both QUIC-5 and PEARLS in logistic regressions in which continuous scores for both were concurrently included as predictors of youth mental health problems and somatic symptoms (Fig. 3 and Supplementary Tables 5 and 6). For most mental and somatic health diagnoses examined, both PEARLS and QUIC-5 provided unique predictive contributions (given that the other is also in the logistic regression model), and the predictive powers (ORs) were similar. For sleep disorders, QUIC-5 was a notably stronger predictor of the increased probability of a diagnosis than PEARLS (Fig. 3).

**Fig. 4:**
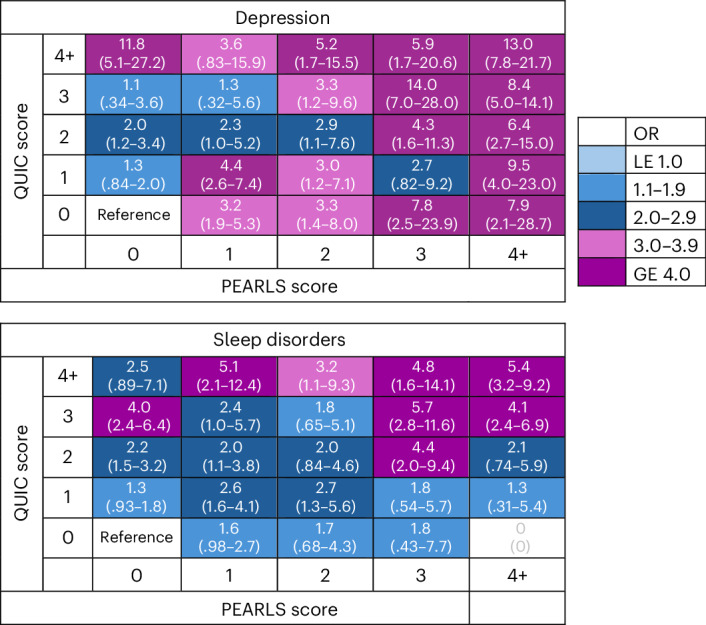
Evaluation of the independent and combined links of ACEs (PEARLS) and unpredictability (QUIC) screens. ORs and 95% CIs are shown. If the lower end of the CI is greater than 1, this indicates a statistically significant increased risk. Examples shown here are for youth self-report. Results for both youth and caregiver reports for all diagnoses can be found in Supplementary Figs. 1 and 2. LE, less than; GE, greater than.

Indeed, QUIC-5 identified a significant risk for mental health problems that was not captured by PEARLS screen alone, as uncovered with the use of the second independent analytic approach: taking depression as an example, the ORs for this diagnosis associated with all possible combinations of QUIC-5 and PEARLS scores were calculated, generating a ‘heat map’. As shown in Fig. 4, top, children with a score of 4 or more on PEARLS had a significantly increased risk of depression: The OR for a PEARLS score of 4 or more and a QUIC-5 score of zero was 7.9, consistent with prevailing knowledge about the contribution of ACEs to subsequent depression. Surprisingly, for the subpopulation (50 children) with PEARLS score of zero and a high QUIC-5 score (4+), the risk for developing depression was 11.7-fold higher compared with the risk of a child who scored a zero on both screens. This fact indicates that the QUIC-5 identifies children at risk for depression that would be missed by the use of current ACEs tools alone. It also bolsters the idea that, independent of other ACEs, unpredictability is a potent harbinger of mental health problems.

This observation was not unique to depression. For example, a child with a high score on PEARLS (4 or more) and with a score of 1 on QUIC-5 was not at increased risk of a sleep disorder (Fig. 4, bottom). Similarly, the increased risk for a sleep disorder generated by a high QUIC-5 score with low PEARLS score (0) was not significant. However, a child with both high PEARLS and QUIC-5 scores (that is, 3 or 4+ on both) was significantly more likely to have a sleep disorder diagnosis than a child with zero scores. Heat maps for all diagnoses as well as the numbers of children who fall in each cell can be found in Supplementary Figs. 1 and 2.

## Discussion

The current studies, conducted with more than 29,000 children at 19 pediatric clinics, have yielded several findings. First, large-scale, systematic screening for ACEs and for early-life unpredictability is feasible in busy primary care settings within a broad range of communities. Throughout these communities, exposure to adversity measured with both QUIC-5 and PEARLS is prevalent even among the youngest children and increases with age. In comparing the two screens, QUIC-5 predicts mental and physical health outcomes at least as well, and in some cases better, than current ACEs screens. Crucially, for some mental health problems (for example depression, sleep disorders), QUIC-5 identifies risk that is not captured by PEARLS screen alone. Because unpredictability of early-life experiences might be amenable to intervention, recognizing its impact on children’s mental health outcomes is a critical prerequisite for devising potential intervention and mitigation approaches.

Orange County, California, USA, the location of the current studies, is home to over 3 million people. CHOC is the dominant healthcare provider in the county. Children receiving well-child and healthcare at the large network of CHOC clinics, and thus included in the current studies, are fully reflective of the county and state’s population and represent a broad range of socioeconomic, ethnic and cultural backgrounds^41^. In addition, the questionnaires were administered during all well-child visits at all clinics, eliminating a potential bias for illness or ‘convenience’ samples. These facts suggest that both the feasibility of the screening and the associations we observed with child health outcomes are likely to be broadly applicable.

As expected based on a robust literature^1–5,8–15,19,42^, exposure to more ACEs captured by screening in pediatric primary care clinics exhibited a graded relationship with risk of a mental health diagnosis. These clear associations have been the impetus for addressing ACEs, aiming to either prevent abuse and neglect^43^ or mitigate their consequences^8–15,44,45^. Prevention of ACEs is a daunting task, and studies aiming to provide fiscal support to families with infants have only been partially successful^43,46^. Alternative approaches have included interventions to combat the effects of ACEs on neurodevelopment or on manifestations of altered stress-responses, anxiety and other mental and physical health issues triggered by ACEs^10,47–53^.

Here, we identify unpredictability of a child’s parental and environmental input as an actionable ACE: introducing predictability into a child’s world may exert protective influences on child development and buffer children from adversity^54,55^. For example, in families experiencing poverty, parental substance use disorders or divorce, family routines predict child resilience^56–58^. Furthermore, during the coronavirus disease 2019 pandemic, a significant stressor that had major impacts on children’s mental health, those children living in families that maintained predictable routines were largely spared the increased oppositional behavior and externalizing symptoms inflicted by pandemic-related disruption on other preschoolers^55^.

How unpredictability influences brain maturation, leading to vulnerability to mental health problems^59,60^, is not fully resolved. For established ACEs, alterations in maturation of specific brain networks (for example, amygdala–prefrontal cortex^6,61–64^) have been reported, and the specific alterations may depend on the dimensions of the ACES and their timing^4,21,63,65^. Altered connectivity of distinct brain nodes has also been shown for unpredictability, including major connections between temporal and frontal regions, and functional responses of hippocampus and amygdala to novelty^66–68^. Although information about the impact of ACEs in children has been accumulating for decades, our understanding of how unpredictability may interfere with the fine-tuning of functional connectivity of the developing brain, leading to symptoms of mental health problems, is in its infancy^21^. A plausible hypothesis, being tested in experimental animal models^21,25^, suggests that predictable sequences of parental and family signals to the child, manifesting as structure in family life and consistent responses and behaviors from parents, may be important as they activate—and thus promote healthy maturation of—brain circuits involved in reward and safety^21^. This creates a solid, foundation for exploration and healthy development^7,29,69^.

For the majority of child health outcomes examined, the ACEs screen and QUIC-5 provide similar degrees of association (Fig. 2). However, the QUIC provides critical additional power to detect risk (Figs. 3 and 4). For sleep disorders, for example, QUIC-5 was superior in risk identification compared with PEARLS. For depression, the contribution of a high QUIC-5 score to the risk of diagnosis (a roughly 12-fold increase with a QUIC-5 score of 4 or above) is striking (Fig. 4 and Supplementary Fig. 1). It is notable that this large effect size is present among individuals with minimal ‘typical’ ACEs, that is, with low scores on PEARLS. These individuals would thus not be identified as high-risk by current screens, potentially leading to missed opportunities for early intervention. The association of unpredictability with sleep disorders is not entirely surprising as optimal sleep is partially dependent on predictable family structure^45^. Together, these findings highlight the value of delineating both the shared and unique contributions of different forms of early-life adversity to individual mental illness. This paves the path for a more granular understanding of individual exposures and the mechanisms through which those exposures operate.

Indeed, in the case of unpredictable childhood experiences, there are multiple opportunities for prevention and intervention, including the prospect of multilevel intervention^54,69^. These range from the level of the individual (for example, altering caregiver attitudes regarding the importance of predictability), to the family systems level (for example, implementing family routines), to the public policy level (for example, adoption of Fair Work Week regulations that address precarious parental work schedules)^54^. Addressing the full range of ACEs should be a top priority to improve the well-being of children and families.

A limitation of the study lies in the use of ORs, which describe relative but not absolute risk for any adverse outcome. Second, we rely on child health diagnoses derived from electronic health records. Given the cross-sectional study design, it is not possible to disentangle the temporal relations between exposures and diagnoses. Further, we are unable to probe the associations between exposures and subclinical symptom profiles, which are highly likely. Future studies will explore how structural and social determinants of health influence exposures to unpredictability and their associations with mental health. This is of particular importance because structural determinants of both ACEs and unpredictability are not evenly distributed and individuals of historically and currently marginalized and systematically excluded backgrounds are at disproportionate risk for these exposures^70–72^.

In summary, we demonstrate that large-scale systematic screening for both ACEs and early-life unpredictability is feasible in primary care clinics serving a broad range of communities. Exposures to adversities detected by these distinct instruments are prevalent even among the youngest children, and both screens predict mental health outcomes and are complementary. Notably, QUIC-5 portends child outcomes and identifies risk for mental health problems that is not captured by the ACEs screen. These findings suggest that adding a screen for unpredictability will^1^ identify children at risk that will be otherwise missed^2^ and may provide an opportunity for monitoring and counseling intervention in the pediatric primary care setting, with the potential to make meaningful positive impacts on children’s mental health.

## Methods

### Study setting and participants

This study took place in 19 pediatric primary care clinics affiliated with CHOC that serve a diverse community of children in Orange County, California, USA (see Table 1 for an overview of demographics). Our research complied with all relevant ethical regulations and was approved by the Children’s Hospital of Orange County In-House Institutional Review Board (#2109123). The primary care clinics implemented routine ACEs screening for all children at their annual well-child visits starting in 2020. Supported by funding from the California Initiative to Advance Precision Medicine^73^, we initiated an optional screening for unpredictability in 2021, and the current study analyzes screens obtained during 2021–2024. After appropriate consent and assent procedures, caregivers filled out both screens, providing information about their child, and the screens were also administered to children aged 12 years and over. Inclusion criteria for the study included: (1) completion of the QUIC-5 screen, (2) child aged 0–17 years and (3) English or Spanish language preference. Here, we present data for the first 29,861 children screened for both ACEs and unpredictability.

### Assessment of ACEs

ACEs were assessed with the face-valid PEARLS^74^. Following current state recommendations, we examined the score for part 1, which focuses on ten ACEs yielding a potential score of 0 to 10. Current protocols at the CHOC pediatric primary care clinics utilize the aggregated or deidentified version of PEARLS in which the respondent provides a count of the number of items positively endorsed without specification of the individual items contributing to these scores. PEARLS scores are then made available in electronic health records.

### Assessment of unpredictability

Unpredictability was assessed with QUIC-5^40,75,76^ (Supplementary Table 7) an abbreviated version of the full-length QUIC, which broadly assesses unpredictability in a child’s social, emotional and physical environments. At the well-child visit, caregiver and child (based on age) were given the opportunity to complete the unpredictability screen. The QUIC-5, on which scores range from 0 to 5, is correlated on average 0.84 with the full-length QUIC^40,75^. Further, validation work in four independent cohorts that spanned gender, socioeconomic groups, multiple languages and ages from 11 to 70 years showed that QUIC-5 predicted mental health outcomes with virtually identical effect sizes to the full-length QUIC.

### Mental health and somatic symptoms

Mental health conditions and somatic symptoms that have strong stress-related and behavioral components and have been previously identified as common outcomes of exposure to early-life adversity in pediatric populations were selected for evaluation^1–8,14,15,42,77^. The presence or absence of the following conditions in each child’s electronic medical record before or concurrent with the well-child visit at which the screen was conducted were obtained: depression, externalizing problems, sleep disorders, anxiety, headache and abdominal pain. Specific International Classification of Diseases (ICD)-10 codes for each diagnosis and the incidence of each diagnosis in Supplementary Table 8.

### Analytic approaches

Analyses were conducted in IBM SPSS version 31. For both QUIC-5 and PEARLS, separate binary logistic regressions were conducted to examine the associations between caregiver and youth reports with mental health outcomes. In these regressions, scores on both screens were categorized as 0, 1, 2, 3 and 4+. To consider the possibility that youth and caregivers reports were incongruent and could differ in predictive power, we also analyzed separately the caregiver and child questionnaires that we obtained for participants aged 12 and older: we examined the distributions of caregiver and youth endorsements on PEARLS and QUIC-5, examined the concordance by item on the QUIC (for which item-level data were available) and performed bivariate correlations to determine the degree of association between youth and caregiver reports for each screen.

We then computed the correlation between PEARLS and QUIC-5, and, to determine whether the QUIC adds predictive power to PEARLS, we used two approaches: (1) QUIC and PEARLS scores (continuous) were entered simultaneously into binary logistic regressions predicting mental health and somatic symptoms outcomes; and (2) in a second set of binary logistic regressions, we examined the independent contributions of all possible combinations of QUIC (0, 1, 2, 3 and 4+) and PEARLS scores (0, 1, 2, 3 and 4+) to these outcomes, generating a ‘heat map’ and setting a score of 0 on each screen as reference. All regression models adjusted for child gender and age (with both linear and quadratic terms considered, as appropriate), and determination of statistical significance was two-sided.

### Reporting summary

Further information on research design is available in the Nature Portfolio Reporting Summary linked to this article.

## Supplementary information

Reporting Summary

Supplementary Fig. 1 Combined predictive contributions (youth self-report). Data summary of the combined and unique predictive contributions of PEARLS and QUIC-5 for youth self-report.

Supplementary Fig. 2 Combined predictive contributions (caregiver report). Data summary of the combined and unique predictive contributions of PEARLS and QUIC-5 for caregiver report.

Supplementary Table 1 Caregiver reports predict child mental health. Caregiver report on PEARLS (*n* = 20,261) and QUIC (*n* = 26,804) screens predict child mental health conditions.

Supplementary Table 2 Youth self-reports predict mental health. Youth self-report on PEARLS (*n* = 8,906) and QUIC (*n* = 9,870) screens predict mental health conditions.

Supplementary Table 3 Caregiver reports predict child mental health after adjustment for socioeconomic status. Caregiver report on PEARLS (*n* = 20,261) and QUIC (*n* = 26,804) screens predict child mental health conditions adjusting for socioeconomic status.

Supplementary Table 4 Youth self-reports predict mental health after adjustment for socioeconomic status. Youth self-report on PEARLS (*n* = 8,906) and QUIC (*n* = 9,870) screens predict mental health conditions after adjusting for socioeconomic status.

Supplementary Table 5 Independent contributions of ACEs and unpredictability to child mental health (caregiver). Binary logistic regressions with PEARLS and QUIC caregiver scores (continuous) concurrently entered as predictors (*n* = 18,486).

Supplementary Table 6 Independent contributions of ACEs and unpredictability to child mental health (youth self-report). Binary logistic regressions with PEARLS and QUIC youth scores (continuous) concurrently entered as predictors (*n* = 7,149).

Supplementary Table 7 QUIC-5 Items. The Questionnaire on Unpredictability in Childhood brief version (QUIC-5)^40^.

Supplementary Table 8 Incidence of child health conditions. Incidence of child mental and somatic health diagnoses (ICD codes used for defining diagnoses shown in parentheses).

## Data Availability

De-identified data will be shared with investigators upon receipt of well-justified request because these involve clinical diagnoses of a sensitive population (children). Requests should be submitted to one of the corresponding authors, L.M.G., at lglynn@chapman.edu, and the reply will be had within 2 weeks.

## Code availability

There was no code available for this study.
