# Supplementary material for: Unpredictability is a childhood adversity that contributes to mental health problems
Source: Nat Ment Health. 2026 Jul 21;4(8):1219–26. doi: 10.1038/s44220-026-00681-x (PMC13441875; doi:10.1038/s44220-026-00681-x)
Supplement: Supplementary file 1 [file 44220_2026_681_MOESM10_ESM.pdf]

Table S7. The Questionnaire of Unpredictability in Childhood brief version (QUIC-5). From Lindert et al., 2022, *Frontiers in Psychology*. 10.3389/fpsyg.2022.971350.

Child/Teen Self-Report

This set of questions asks about your family life. When we say parents, we mean your parents and whoever else takes care of you most of the time (for example, biological parents, step-parents, grandparents, foster parents). This could be one person, or this could be more than one person.

We are going to ask about your experiences from birth to age 18 (or your whole life, if you are less than 18 years old). These answers should be based on your own memories, not on things you later learned from your parents or others.

Please answer these questions based on how things are most of the time.

	Yes	No
**1. Before I was 12 years old, I had a bedtime routine (for example, my parents tucked me in, my parents read me a book, I took a bath).**	☐	☐
**2. At least one of my parents was unpredictable.**	☐	☐
**3. One of my parents could go from calm to furious (very angry) in an instant.**	☐	☐
**4. My parents had a stable relationship with each other.**	☐	☐
**5. In my house, things I needed were often misplaced so that I could not find them.**	☐	☐

Parent/Caregiver Report

This set of questions asks about your child's experiences. When we say parents, we mean you and whoever else in your child's life fills that role for your child (e.g., biological parents, step-parents, grandparents, foster parents). This could be one person, or this could be multiple people.

We are going to ask about your child's experiences from birth to age 18 (or your child's life until now, if he/she is less than 18 years old).

Please answer these questions based on your child's typical or average experiences.

	Yes	No
**6. Before age 12, my child had a bedtime routine (e.g., was tucked in, parents read him/her a book, took a bath).**	☐	☐
**7. One or more of my child's parents was unpredictable.**	☐	☐

**8. One or more of my child's parents could go from calm to furious in an instant.**	☐	☐
**9. My child's parents had a stable relationship with each other.**	☐	☐
**10. In my child's house, things they needed were often misplaced so that we could not find them.**	☐	☐
