# Supplementary material for: Unpredictability is a childhood adversity that contributes to mental health problems
Source: Nat Ment Health. 2026 Jul 21;4(8):1219–26. doi: 10.1038/s44220-026-00681-x (PMC13441875; doi:10.1038/s44220-026-00681-x)
Supplement: Supplementary file 2 [file 44220_2026_681_MOESM11_ESM.pdf]

**Table S8.** Incidence of child mental and somatic health diagnoses (ICD codes used for defining diagnoses shown in parentheses).

| Diagnosis | | n | % |
|---|---|---|---|
| **Depression**<br>(F32.0-F32.5, F32.81, F32.89, F32.9, F33.0-F33.3, F33.40-F33.42, F33.8, F33.9, F34.1, F348, F34.81) | | | |
| | **yes** | 545 | 1.9 |
| | **no** | 28,760 | 98.1 |
| **Anxiety**<br>(F40.00-F40.02, F40.10, F40.11, F40.210, F40.218, F40.220, F40.228, F40.230-F40.233, F40.240-F40.243, F40.248, F40.290, F40.291, F40.298, F40.8, F40.9, F41.0-F41.3, F41.8, F41.9, F93.0, F94.0) | | | |
| | **yes** | 1,890 | 6.4 |
| | **no** | 27,495 | 96.3 |
| **Sleep Disorders**<br>(G47) | | | |
| | **yes** | 1,812 | 6.2 |
| | **no** | 27,493 | 93.8 |
| **Externalizing Problems**<br>(F63.0-F63.2, F63.81, F63.89, F63.9, F91.0-F91.3, F91.8, F91.9) | | | |
| | **yes** | 494 | 1.7 |
| | **no** | 28,811 | 98.3 |
| **Headache**<br>(G43, G44, R51) | | | |
| | **yes** | 2,562 | 8.7 |
| | **no** | 26,743 | 91.3 |
| **Abdominal Pain**<br>(R10) | | | |
| | **yes** | 4,143 | 14.1 |
| | **no** | 25,612 | 85.9 |
