# Supplementary material for: Unpredictability is a childhood adversity that contributes to mental health problems
Source: Nat Ment Health. 2026 Jul 21;4(8):1219–26. doi: 10.1038/s44220-026-00681-x (PMC13441875; doi:10.1038/s44220-026-00681-x)
Supplement: Supplementary file 4 [file 44220_2026_681_MOESM4_ESM.pdf]

Table S1. Caregiver report on PEARLS (n = 20,261) and QUIC (n = 26,804) screens predict child mental health conditions.

	PEARLS					QUIC-5			
Diagnosis	Score	Adjusted Odds Ratio	95% Confidence Interval	p-value		Score	Adjusted Odds Ratio	95% confidence Interval	p-value
Depression	0	1.0	Referent			0	1.0	Referent	
	1	2.1	(1.6-2.9)	<.001		1	1.5	(1.2-1.9)	<.001
	2	3.3	(2.3-4.8)	<.001		2	2.3	(1.7-3.0)	<.001
	3	3.4	(2.1-5.5)	<.001		3	2.5	(1.7-3.5)	<.001
	4 or more	5.4	(3.9-7.4)	<.001		4 or more	3.2	(1.8-5.7)	<.001
Anxiety	0	1.0	Referent			0	1.0	Referent	
	1	1.6	(1.3-1.9)	<.001		1	1.2	(1.1-1.4)	.01
	2	2.1	(1.7-2.7)	<.001		2	1.5	(1.2-1.7)	<.001
	3	1.6	(1.2-2.3)	<.001		3	1.9	(1.6-2.4)	<.001
	4 or more	3.1	(2.5-3.8)	<.001		4 or more	1.7	(1.2-2.6)	.01
Sleep Disorders	0	1.0	Referent			0	1.0	Referent	
	1	1.4	(1.2-1.7)	<.001		1	1.3	(1.1-1.4)	<.001
	2	1.7	(1.3-2.2)	<.001		2	1.7	(1.4-1.9)	<.001
	3	2.1	(1.5-2.8)	<.001		3	2.1	(1.7-2.6)	<.001
	4 or more	1.9	(1.5-2.5)	<.001		4 or more	2.5	(1.7-3.6)	<.001
Externalizing Problems	0	1.0	Referent			0	1.0	Referent	
	1	2.2	(1.6-2.9)	<.001		1	1.4	(1.1-1.7)	.01
	2	1.8	(1.1-2.9)	.02		2	2.0	(1.5-2.7)	<.001
	3	2.7	(1.6-4.6)	<.001		3	3.1	(2.2-4.3)	<.001
	4 or more	4.5	(3.1-6.5)	<.001		4 or more	3.6	(2.0-6.5)	<.001
Headache	0	1.0	Referent			0	1.0	Referent	
	1	1.3	(1.2-1.6)	<.001		1	1.2	(1.1-1.4)	<.001
	2	1.5	(1.2-1.9)	<.001		2	1.5	(1.3-1.7)	<.001
	3	1.2	(.87-1.6)	.27		3	2.0	(1.7-2.4)	<.001
	4 or more	1.9	(1.5-2.3)	<.001		4 or more	1.9	(1.4-2.7)	<.001
Abdominal Pain	0	1.0	Referent			0	1.0	Referent	
	1	1.2	(1.0-1.3)	.01		1	1.4	(1.3-1.5)	<.001
	2	1.3	(1.1-1.6)	<.001		2	1.4	(1.3-1.6)	<.001
	3	1.4	(1.1-1.7)	.02		3	1.5	(1.3-1.8)	<.001
	4 or more	1.6	(1.3-2.0)	<.001		4 or more	1.6	(1.1-2.1)	.01

Note: Odds ratio estimates adjusted for child age, child age* and sex.
