# Supplementary material for: Unpredictability is a childhood adversity that contributes to mental health problems
Source: Nat Ment Health. 2026 Jul 21;4(8):1219–26. doi: 10.1038/s44220-026-00681-x (PMC13441875; doi:10.1038/s44220-026-00681-x)
Supplement: Supplementary file 5 [file 44220_2026_681_MOESM5_ESM.pdf]

Table S2. Youth self-report on PEARLS (n = 8,906) and QUIC (n = 9,870) screens predict mental health conditions.

	PEARLS					QUIC-5			
Diagnosis	Score	Adjusted Odds Ratio	95% Confidence Interval	p-value		Score	Adjusted Odds Ratio	95% confidence Interval	p-value
Depression	0	1.0	Referent			0	1.0	Referent	
	1	2.7	(2.0-3.6)	<.001		1	1.4	(1.1-1.9)	.01
	2	2.8	(1.9-4.2)	<.001		2	2.1	(1.6-2.9)	<.001
	3	5.1	(3.4-7.5)	<.001		3	3.7	(2.7-5.0)	<.001
	4 or more	7.2	(5.3-9.7)	<.001		4 or more	6.1	(4.3-8.7)	<.001
Anxiety	0	1.0	Referent			0	1.0	Referent	
	1	1.7	(1.4-2.0)	<.001		1	1.2	(.99-1.4)	.07
	2	1.9	(1.5-2.5)	<.001		2	1.6	(1.3-1.9)	<.001
	3	3.0	(2.3-4.1)	<.001		3	2.0	(1.6-2.5)	<.001
	4 or more	3.7	(2.9-4.7)	<.001		4 or more	3.3	(2.5-4.3)	<.001
Sleep Disorders	0	1.0	Referent			0	1.0	Referent	
	1	1.6	(1.2-2.1)	<.001		1	1.3	(1.0-1.6)	.03
	2	1.6	(1.1-2.3)	.01		2	1.9	(1.5-2.4)	<.001
	3	2.7	(1.9-4.0)	<.001		3	2.6	(2.0-3.5)	<.001
	4 or more	2.6	(1.9-3.6)	<.001		4 or more	3.0	(2.2-4.3)	<.001
Externalizing Problems	0	1.0	Referent			0	1.0	Referent	
	1	2.3	(1.4-3.6)	<.001		1	1.7	(1.1-2.5)	.02
	2	2.7	(1.5-5.1)	<.001		2	2.7	(1.7-4.3)	<.001
	3	4.7	(2.5-8.8)	<.001		3	3.3	(2.0-5.6)	<.001
	4 or more	4.9	(2.9-8.4)	<.001		4 or more	4.7	(2.5-8.7)	<.001
Headache	0	1.0	Referent			0	1.0	Referent	
	1	1.5	(1.2-1.8)	<.001		1	1.2	(1.2-1.6)	<.001
	2	1.4	(1.1-1.8)	.01		2	1.3	(1.3-1.8)	<.001
	3	1.6	(1.1-2.1)	.01		3	1.4	(1.4-2.2)	<.001
	4 or more	1.7	(1.4-2.3)	<.001		4 or more	1.6	(1.6-2.7)	<.001
Abdominal Pain	0	1.0	Referent			0	1.0	Referent	
	1	1.4	(1.2-1.6)	.01		1	1.4	(.96-1.0)	<.001
	2	1.4	(1.2-1.8)	<.001		2	1.6	(1.4-1.9)	<.001
	3	1.5	(1.1-2.0)	.14		3	1.6	(1.3-1.9)	<.001
	4 or more	1.7	(1.3-2.1)	<.001		4 or more	2.0	(1.6-2.6)	<.001

Note: Odds ratio estimates adjusted for child age and sex.
