# Supplementary material for: Unpredictability is a childhood adversity that contributes to mental health problems
Source: Nat Ment Health. 2026 Jul 21;4(8):1219–26. doi: 10.1038/s44220-026-00681-x (PMC13441875; doi:10.1038/s44220-026-00681-x)
Supplement: Supplementary file 6 [file 44220_2026_681_MOESM6_ESM.pdf]

**Table S3**. Caregiver report on PEARLS (n = 20,261) and QUIC (n = 26,804) screens predict child mental health conditions adjusting for socioeconomic status.									
	**PEARLS**					**QUIC-5**			
Diagnosis	Score	Adjusted Odds Ratio	95% Confidence Interval	p-value		Score	Adjusted Odds Ratio	95% confidence Interval	p-value
Depression	0	1.0	Referent			0	1.0	Referent	
	1	2.1	(1.6-2.8)	<.001		1	1.1	(.99-1.6)	.07
	2	3.3	(2.2-4.8)	<.001		2	1.3	(1.1-1.6)	<.001
	3	3.3	(2.0-5.3)	<.001		3	1.7	(1.4-2.2)	<.001
	4 or more	4.8	(3.5-6.7)	<.001		4 or more	1.5	(1.0-2.2)	.05
Anxiety	0	1.0	Referent			0	1.0	Referent	
	1	1.5	(1.3-1.8)	<.001		1	1.1	(.98-1.3)	.11
	2	2.1	(1.6-2.6)	<.001		2	1.5	(1.2-1.8)	<.001
	3	1.6	(1.2-2.3)	.004		3	1.8	(1.4-2.3)	<.001
	4 or more	2.9	(2.3-3.6)	<.001		4 or more	2.8	(2.2-3.7)	<.001
Sleep Disorders	0	1.0	Referent			0	1.0	Referent	
	1	1.4	(1.1-1.6)	<.001		1	1.1	(.96-1.2)	.20
	2	1.6	(1.2-2.1)	<.001		2	1.4	(1.1-1.6)	<.001
	3	2.0	(1.4-2.7)	<.001		3	1.7	(1.4-2.2)	<.001
	4 or more	1.7	(1.3-2.2)	<.001		4 or more	2.0	(1.4-3.0)	<.001
Externalizing Problems	0	1.0	Referent			0	1.0	Referent	
	1	2.1	(1.6-2.8)	<.001		1	1.2	(.93-1.5)	.18
	2	1.8	(1.1-2.9)	.02		2	1.7	(1.2-2.2)	<.001
	3	2.5	(2.5-1.5)	<.001		3	2.6	(1.8-3.6)	<.001
	4 or more	4.0	(2.8-5.7)	<.001		4 or more	2.9	(1.6-5.3)	<.001
Headache	0	1.0	Referent			0	1.0	Referent	
	1	1.3	(1.1-1.6)	<.001		1	1.0	(.93-1.2)	.43
	2	1.4	(1.2-1.8)	.001		2	1.2	(1.0-1.4)	.01
	3	1.1	(.79-1.5)	.59		3	1.6	(1.4-2.0)	<.001
	4 or more	1.7	(1.3-2.1)	<.001		4 or more	1.5	(1.1-2.2)	.01
Abdominal Pain	0	1.0	Referent			0	1.0	Referent	
	1	1.1	(1.0-1.3)	.04		1	1.2	(1.1-1.3)	<.001
	2	1.2	(1.0-1.5)	.04		2	1.1	(1.0-1.3)	.03
	3	1.3	(.98-1.6)	.07		3	1.3	(1.1-1.5)	.005
	4 or more	1.4	(1.2-1.7)	<.001		4 or more	1.3	(.92-1.7)	.16

Note: Odds ratio estimates adjusted for insurance type (public versus private), child age, child age* and sex.
