# Supplementary material for: Unpredictability is a childhood adversity that contributes to mental health problems
Source: Nat Ment Health. 2026 Jul 21;4(8):1219–26. doi: 10.1038/s44220-026-00681-x (PMC13441875; doi:10.1038/s44220-026-00681-x)
Supplement: Supplementary file 7 [file 44220_2026_681_MOESM7_ESM.pdf]

Table S4. Youth self-report on PEARLS (n = 8,906) and QUIC (n = 9,870) screens predict mental health conditions after adjusting for socioeconomic status.

	PEARLS					QUIC-5			
Diagnosis	Score	Adjusted Odds Ratio	95% Confidence Interval	p-value		Score	Adjusted Odds Ratio	95% confidence Interval	p-value
Depression	0	1.0	Referent			0	1.0	Referent	
	1	2.6	(1.9-3.5)	<.001		1	1.2	(.93-1.6)	.15
	2	2.5	(1.7-3.7)	<.001		2	1.8	(1.4-2.5)	<.001
	3	4.8	(3.2-7.1)	<.001		3	3.2	(2.3-4.4)	<.001
	4 or more	6.6	(4.8-8.9)	<.001		4 or more	4.9	(3.5-7.0)	<.001
Anxiety	0	1.0	Referent			0	1.0	Referent	
	1	1.6	(1.3-2.0)	<.001		1	1.1	(.94-1.3)	.20
	2	1.8	(1.4-2.4)	<.001		2	1.5	(1.2-1.8)	<.001
	3	3.0	(2.2-4.0)	<.001		3	1.8	(1.4-2.3)	<.001
	4 or more	3.5	(2.8-4.5)	<.001		4 or more	2.8	(2.2-3.7)	<.001
Sleep Disorders	0	1.0	Referent			0	1.0	Referent	
	1	1.5	(1.2-2.0)	<.001		1	1.1	(.90-1.4)	.30
	2	1.5	(1.0-2.1)	.04		2	1.6	(1.2-2.0)	<.001
	3	2.5	(1.7-3.6)	<.001		3	2.1	(1.6-2.8)	<.001
	4 or more	2.3	(1.7-3.2)	<.001		4 or more	2.4	(1.7-3.7)	<.001
Externalizing Problems	0	1.0	Referent			0	1.0	Referent	
	1	2.0	(1.3-3.2)	.003		1	1.5	(.98-2.3)	.06
	2	2.3	(1.2-4.5)	<.001		2	2.3	(1.5-3.7)	<.001
	3	4.3	(2.3-8.1)	<.001		3	2.8	(1.6-4.8)	<.001
	4 or more	4.3	(2.5-7.3)	<.001		4 or more	3.1	(1.6-6.1)	<.001
Headache	0	1.0	Referent			0	1.0	Referent	
	1	1.4	(1.2-1.7)	<.001		1	1.2	(1.0-1.4)	.03
	2	1.3	(.97-1.6)	.09		2	1.2	(1.0-1.5)	<.001
	3	1.4	(1.0-2.0)	.03		3	1.4	(1.2-2.1)	<.001
	4 or more	1.5	(1.2-1.9)	.001		4 or more	1.6	(1.2-2.1)	<.001
Abdominal Pain	0	1.0	Referent			0	1.0	Referent	
	1	1.3	(1.1-1.5)	.002		1	1.2	(1.1-1.4)	.01
	2	1.3	(1.1-1.7)	.02		2	1.4	(1.2-1.6)	<.001
	3	1.5	(1.1-2.0)	.007		3	1.3	(1.1-1.6)	<.001
	4 or more	1.5	(1.2-1.9)	<.001		4 or more	1.6	(1.3-2.1)	<.001

Note: Odds ratio estimates adjusted for insurance type (public versus private), child age and sex.
