# Supplementary material for: Unpredictability is a childhood adversity that contributes to mental health problems
Source: Nat Ment Health. 2026 Jul 21;4(8):1219–26. doi: 10.1038/s44220-026-00681-x (PMC13441875; doi:10.1038/s44220-026-00681-x)
Supplement: Supplementary file 8 [file 44220_2026_681_MOESM8_ESM.pdf]

**Table S5**. Binary logistic regressions with PEARLS and QUIC caregiver scores (continuous) concurrently entered as predictors (n = 18,486).				
Diagnosis	Score	Adjusted Odds Ratio	95% Confidence Interval	p-value
Depression				
	PEARLS	1.3	(1.2-1.4)	<.001
	QUIC	1.1	(.98-1.2)	.12
Anxiety				
	PEARLS	1.2	(1.1-1.2)	<.001
	QUIC	1.1	(1.0-1.2)	<.001
Sleep Disorders				
	PEARLS	1.1	(1.0-1.1)	.01
	QUIC	1.2	(1.1-1.3)	<.001
Externalizing Problems				
	PEARLS	1.2	(1.1-1.3)	<.001
	QUIC	1.2	(1.1-1.4)	<.001
Headache				
	PEARLS	1.1	(1.0-1.1)	.02
	QUIC	1.2	(1.1-1.2)	<.001
Abdominal Pain				
	PEARLS	1.0	(1.0-1.1)	.01
	QUIC	1.1	(1.1-1.2)	<.001
