# Supplementary material for: Unpredictability is a childhood adversity that contributes to mental health problems
Source: Nat Ment Health. 2026 Jul 21;4(8):1219–26. doi: 10.1038/s44220-026-00681-x (PMC13441875; doi:10.1038/s44220-026-00681-x)
Supplement: Supplementary file 9 [file 44220_2026_681_MOESM9_ESM.pdf]

Table S6. Binary logistic regressions with PEARLS and QUIC youth scores (continuous) concurrently entered as predictors (n = 7,149).

Diagnosis	Score	Adjusted Odds Ratio	95% Confidence Interval	p-value
Depression				
	PEARLS	1.3	(1.2-1.4)	<.001
	QUIC	1.2	(1.1-1.4)	<.001
Anxiety				
	PEARLS	1.2	(1.1-1.2)	<.001
	QUIC	1.1	(1.1-1.2)	<.001
Sleep Disorders				
	PEARLS	1.1	(1.0-1.1)	.06
	QUIC	1.4	(1.3-1.5)	<.001
Externalizing Problems				
	PEARLS	1.2	(1.1-1.3)	<.001
	QUIC	1.3	(1.1-1.6)	<.001
Headache				
	PEARLS	1.1	(1.0-1.1)	.04
	QUIC	1.2	(1.1-1.2)	<.001
Abdominal Pain				
	PEARLS	1.0	(.98-1.1)	.32
	QUIC	1.2	(1.1-1.2)	<.001
